# Experimental investigation of quantum entropic uncertainty relations for multiple measurements in pure diamond

**DOI:** 10.1038/s41598-017-02424-6

**Published:** 2017-05-31

**Authors:** Jian Xing, Yu-Ran Zhang, Shang Liu, Yan-Chun Chang, Jie-Dong Yue, Heng Fan, Xin-Yu Pan

**Affiliations:** 1 Beijing National Laboratory for Condensed Matter Physics, Institute of Physics, Chinese Academy of Sciences, Beijing, 100190 China; 2 School of Physical Sciences, University of Chinese Academy of Sciences, Beijing, 100190 China; 3 School of Physics, Peking University, Beijing, 100871 China; 4 Collaborative Innovation Center of Quantum Matter, 100190 Beijing, China

## Abstract

One unique feature of quantum mechanics is the Heisenberg uncertainty principle, which states that the outcomes of two incompatible measurements cannot simultaneously achieve arbitrary precision. In an information-theoretic context of quantum information, the uncertainty principle can be formulated as entropic uncertainty relations with two measurements for a quantum bit (qubit) in two-dimensional system. New entropic uncertainty relations are studied for a higher-dimensional quantum state with multiple measurements, and the uncertainty bounds can be tighter than that expected from two measurements settings and cannot result from qubits system with or without a quantum memory. Here we report the first room-temperature experimental testing of the entropic uncertainty relations with three measurements in a natural three-dimensional solid-state system: the nitrogen-vacancy center in pure diamond. The experimental results confirm the entropic uncertainty relations for multiple measurements. Our result represents a more precise demonstrating of the fundamental uncertainty principle of quantum mechanics.

## Introduction

One significant feature of quantum theory that differs from our everyday life experience is the uncertainty principle which was first introduced in 1927 by Heisenberg^1^. The uncertainty relation that bounds the uncertainties about the measurement outcomes of two incompatible observables on one particle can be formulated using the standard deviation. One widely accepted form of this relation is expressed by the Heisenberg-Robertson relation^2^: Δ*R*Δ*S* ≥ |〈[*R*, *S*]〉|/2 where Δ*R* is the standard deviation of an observable *R*. Since this form of relations is state-dependent on the right-hand-side, an improvement of uncertainty relation, in an information-theoretic context, was subsequently proposed and expressed as^3, 4^ *H*(*R*) + *H*(*S*) ≥ log_2_[1/*c*(*R*, *S*)] where *H*(*R*) denotes the Shannon entropy of the probability distribution of the outcomes when *R* is measured and $c(R,S)\equiv {{\rm{\max }}}_{j,k}{|\langle {r}_{j}|{s}_{k}\rangle |}^{2}$ given |*r*_*j*_〉 and |*s*_*k*_〉 the eigenvectors of *R* and *S*, respectively. In the presence of a quantum memory, the uncertainty relation can be generalized as^5^ *H*(*R* | B) + *H*(*S* | B) ≥ log_2_[1/*c*(*R*, *S*)] *H*(A | B) where *H*(*R* | B) denotes the conditional von Neumann entropy. It provides a bound on the uncertainties of the measurement outcomes depending on the entanglement between measured particle A and the quantum memory B and is validated by recent experiments^6, 7^. These results as well as related investigations^8–10^ have been discovered to have many significant applications, such as the security proofs for quantum cryptography^11, 12^, nonlocality^13^ and the separability problem^14^. Besides, in some recent researches, the fundamental reason of uncertainty relations have been investigated extending to more general theories such as thermodynamics^15, 16^ and relativity^17^. It is indicated that the violation of the uncertainty relations would lead to a violation of the second law of thermodynamics.

There are also efforts made to generalize the uncertainty relations to more than two observables^18^ and the entropic uncertainty relations for multiple measurements with general condition are studied theoretically by some of us^19^ and another group^20^. The bounds^19^ for multiple measurements in higher-dimension are tighter than that obtained from two measurements results, so those uncertainty relations provide a more precise description of the uncertainty principle which may highlight the boundary between quantum and classical physics. Thus, the essence of those uncertainty relations can be well demonstrated in a three-dimension quantum system like a spin-1 state for the reasons that they cannot be obtained from the ordinary two measurements setting, and the indivisible quantum system cannot result in nonlocality or entanglement^21^.

In this work, we report the first room-temperature proof-of-principle implementation of the entropic uncertainty relations for multiple measurements^19^ in a solid-state system: the nitrogen-vacancy (NV) center in pure diamond single crystal. An individual NV center can be viewed as a basic unit of a quantum computer and is one of the most promising candidates for quantum information processing (QIP), since many coherent control and manipulation processes have been performed with this system^22–37^. Here, we demonstrate the entropic uncertainty relations for multiple measurements via the triplet ground states of the spin-1 electron spin of a single NV center. Since the entropic uncertainty relations are state dependent, we further investigate different initial states of spin-1 electron spin of a single NV center. We also change the complementarity of three measured observables and verify different types of entropic uncertainty relations for multiple measurements. Moreover, our system is a truly three-level system and has overcome the defects of post-selection in the most common optical systems, which differs from earlier relative works.

## Results

### System description and experimental setup

**Figure 1:**
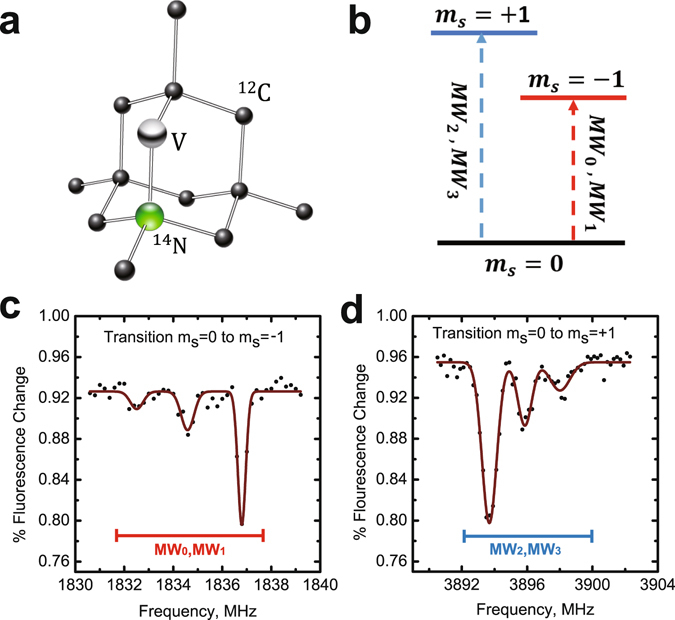
Typical structure of NV center in pure diamond single crystal. (**a**) The NV center consists of a nearest-neighbor pair of a ^14^N atom, which substitutes for a ^12^C atom, and a lattice vacancy (V). (**b**) Three energy levels of the ground state of NV center. The electron spin state is controlled by MW pulses. MW_0_ and MW_2_ indicate MW pulses with a phase of 0, while MW_1_ and MW_3_ indicate MW pulses with a phase of *π*/2. (**c**) ODMR spectra of transition *m*_*s*_ = 0 to *m*_*s*_ = −1. (**d**) ODMR spectra of transition *m*_*s*_ = 0 to *m*_*s*_ = +1.

The electron spin of NV center interacts with the external magnetic field, causing a splitting of the three-energy spin states. The Hamiltonian of a negative charged NV center (NV^−^) in pure diamond under an external magnetic field ***B*** is written as

1 $$\begin{matrix}H & = & {\rm{\Delta }}{S}_{z}^{2}-{\gamma }_{e}{\boldsymbol{B}}\cdot {\boldsymbol{S}}-{\gamma }_{N}{\boldsymbol{B}}\cdot {{\boldsymbol{I}}}^{(N)}-{\gamma }_{c}{\boldsymbol{B}}\cdot \sum _{i}{{\boldsymbol{I}}}_{i}^{(C)}\\  &  & +{A}_{\parallel }^{(N)}{S}_{z}{I}_{z}^{(N)}+{A}_{\perp }^{(N)}{S}_{x}{I}_{x}^{(N)}+{A}_{\perp }^{(N)}{S}_{y}{I}_{y}^{(N)}\\  &  & +{S}_{z}\sum _{i}{{\boldsymbol{A}}}_{i}\cdot {{\boldsymbol{I}}}_{i}^{(C)}\end{matrix}$$

where Δ = 2.87 GHz is the zero-field splitting of the spin-1 ground states. γ_*e*_ = 2.80 × 10^10^ T^−1^s^−1^ and γ_*c*_ = 1.07 × 10^7^ T^−1^s^−1^ are the gyromagnetic ratio of electron spins and ^13^C nuclear spins, respectively. ***A***_*i*_ is the hyperfine tensor for ${{\boldsymbol{I}}}_{i}^{(C)}$. ${A}_{\parallel }^{(N)}$ and ${A}_{\perp }^{(N)}$ are hyperfine constants for ***I*** ^(*N*)^. In this condition, the electron spin couples with the ${{\rm{I}}}_{N}=1({{\rm{m}}}_{{N}_{s}}\pm \mathrm{1,0})$ nuclear spin, so m_*s*_ = −1 level splits into three energy levels with states denoted by the Dirac notation $|{m}_{{N}_{s}},{m}_{s}\rangle$: |1, −1〉, |0, −1〉 and |−1, −1〉. In Fig. 1(e) and (f), each one of the three transitions from an energy level m_*s*_ = 0 to another level m_*s*_ = ±1 indicates a dip in the spectra.

The experiment is implemented with one single NV center in pure diamond (Sumitomo, Nitrogen Concentration <5 ppb). The decoherence of NV centers in this sample is dominated by the nuclear spins of ^13^C atoms. NV centers in diamond are surrounded by randomly distributed ^13^C atoms as the natural abundance of ^13^C is 1.1%. The nuclear spin of the ^13^C atom would interact with NV center electron spin leading to the extra splitting and decoherence. The typical dephasing time (${T}_{2}^{\ast }$) of NV centers in this sample is over 600 ns. For a better manipulation fidelity, we choose the NV center without nearby ^13^C atoms. A permanent magnet is used to apply an external magnetic field on the system and is tuneable in both strength and orientation. Under several circumstance, excited-state level anti-crossing (ESLAC) of the center electron spin is used for the nuclear spin polarization^38^. When the magnetic field is around the ESLAC point (about 507 Gauss), laser driven electron spin polarization would transfer to nearby nuclear spins. In this experiment, the magnet is adjusted to about 370 Gauss as the ^14^N nuclear spin is partially polarized to improve the operation fidelity.

**Figure 2:**
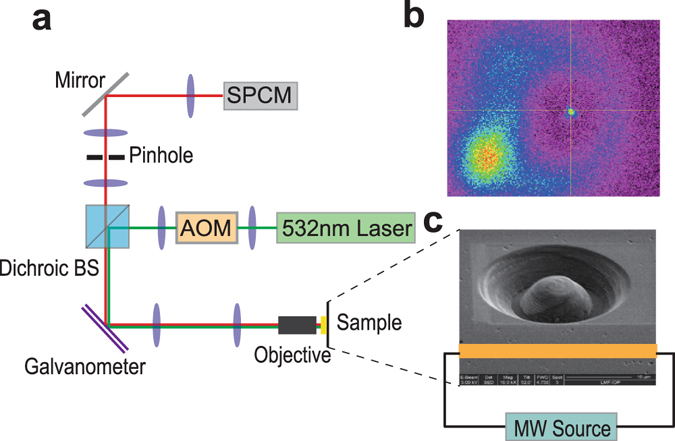
Experimental setup. (**a**) Sketch map of the home-built scanning confocal microscope. A 532 nm Laser beam from laser device is switched by an acoustic optic modulator (AOM) and focused on the sample through a microscope objective. The fluorescence of NV center is collected by the same objective and detected by the single photon counting meter (SPCM). The galvanometer is used to perform an X-Y scan of the sample while the dichroic beam-splitter (BS) is used to split the fluorescence of NV center and Laser. (**b**) Typical fluorescence scanning chart of the SIL and the NV center in it. (**c**) Typical photo of the SIL taken by electron microscope and sketch map of microwave system.

**Figure 3:**
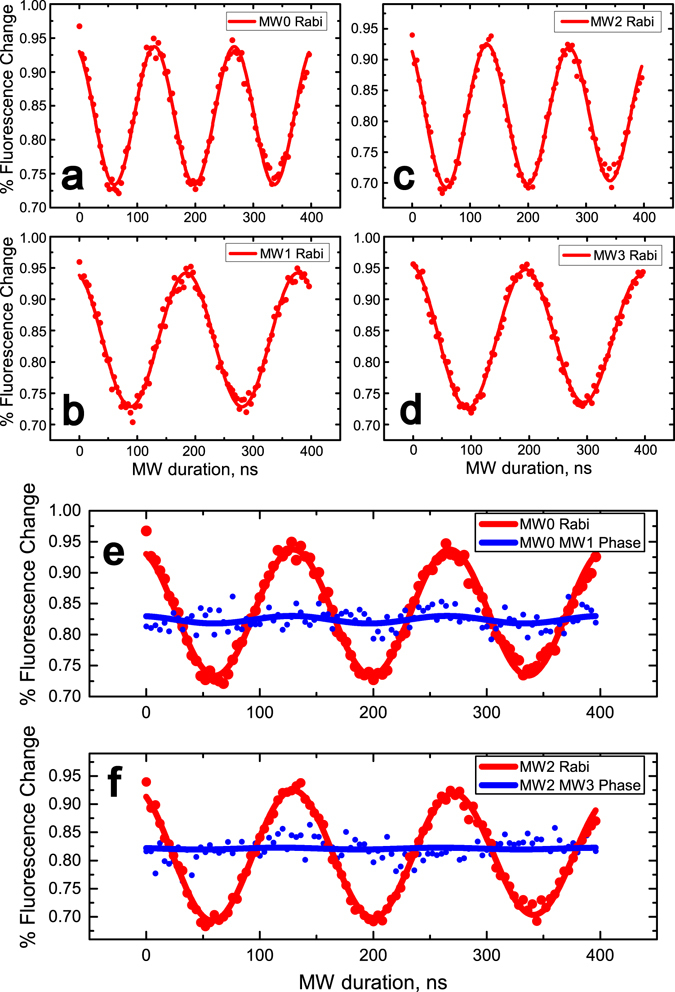
Rabi oscillations carried out by the four MW channels. (**a**) MW_0_. (**b**) MW_1_. (**c**) MW_2_. (**d**) MW_3_. (**e**) Red line shows the Rabi oscillation carried out by MW_0_. Blue line shows the Rabi oscillation carried out by MW_1_ after a MW_0_ $\frac{\pi }{2}$ pulse. (**f**) Red line shows the Rabi oscillation carried out by MW_2_. Blue line shows the Rabi oscillation carried out by MW_3_ after a MW_2_ $\frac{\pi }{2}$ pulse.

Hyperfine spectra of the NV center is obtained by optically detected magnetic resonance (ODMR)^39^ scanning as shown is Fig. 1. A home-built scanning confocal microscope combined with integrated microwave (MW) devices, as shown in Fig. 2, is employed to initialize, manipulate and read out the electron spin state. A 532 nm laser beam from the laser device is switched by an acoustic optic modulator (AOM) and focused on the sample through a microscope objective. The fluorescence of NV center is collected by the same objective and detected by the single photon counting meter (SPCM). The galvanometer is used to perform an X-Y scan of the sample while the dichroic beam-splitter (BS) is used to split the fluorescence of NV center and laser. Resonance microwave is used to control the electron spin state. To enhance the photon collection efficiency, a solid immersion lens (SIL)^40^ is etched above the NV center. A coplanar waveguide (CPW) antenna is deposited close to the SIL to deliver the microwave pulses to the NV center. Typical fluorescence scanning chart of the SIL and the NV center in it is shown in Fig. 2(b). The photo of SIL taken by electron microscope and sketch map of microwave system is also indicated in Fig. 2(c). Four MW channels (MW_0_, MW_1_, MW_2_, MW_3_) controlled by individual RF switches for state and phase controls of the electron spin (Fig. 1) are used in this experiment. MW_1_ and MW_3_ are respectively set to have a *π*/2 phase shift relative to MW_0_ and MW_2_. In Fig. 3(a–d), the Rabi oscillations carried out by the four MW channels are implemented. Figure 3(e) and (f) show that the relative phase between MW_1_ and MW_0_ and the one between MW_3_ and MW_2_ are both *π*/2.

**Figure 4:**
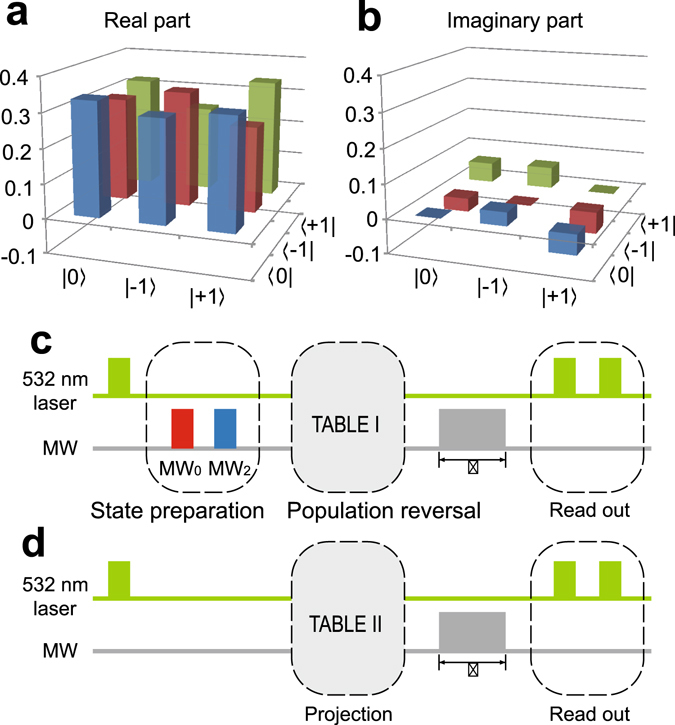
State tomography and pulse sequences for entropy measurement and state tomography. (**a**) Real part of state tomography result of an electron spin superposition state $\frac{1}{\sqrt{3}}(|0\rangle +|-1\rangle +|+1\rangle )$. (**b**) Imaginary part of state tomography result. (**c**) Pulse sequence for state tomography. State preparation is executed by adopting MW_0_ with 26 ns and MW_2_ with 26 ns. Population reversal is implemented by MW pulses shown in Table 1. (**d**) Pulse sequence for generalized entropic uncertainty relations for multiple measurements. The projection scheme is carried out by MW pulses shown in Table 2. The MW pulse whose length is *τ* indicates the Rabi oscillation scheme.

To demonstrate that our system is a truly three-level system which can overcome the defects of post-selection in common optical systems, we plot the state tomography result of an electron spin superposition state $|\psi \rangle =\frac{1}{\sqrt{3}}(|0\rangle +|-1\rangle +|+1\rangle )$ in Fig. 4(a) and (b). See Methods for details. Pulse sequence for state tomography is shown in Fig. 4(c) with MW pulses for different measurement bases shown in Table 1. The fidelity of the experimental result is about 95.35%, which is calculated from $F(\rho )={\rm{Tr}}\sqrt{\sqrt{\sigma }\rho \sqrt{\sigma }}$ with *σ* = |*ψ*〉〈*ψ*|. As a result, our truly three-level system is well suitable for the investigation of generalized entropic uncertainty relations for multiple measurements.

**Table 1:** Eigenvectors for state tomography.

Eigenvector	Population reversal	Rabi
**set 1**		
|0〉	**NA**	**MW**_**0**_
|−1〉	**NA**	**MW**_**0**_
$\sqrt{0.5}(|0\rangle -|-1\rangle )$	**NA**	**MW**_**0**_
$\sqrt{0.5}(|0\rangle -i|-1\rangle )$	**NA**	**MW**_**1**_
**set 2**		
|0〉	**NA**	**MW**_**2**_
|+1〉	**NA**	**MW**_**2**_
$\sqrt{0.5}(|0\rangle -|+1\rangle )$	**NA**	**MW**_**2**_
$\sqrt{0.5}(|0\rangle -i|+1\rangle )$	**NA**	**MW**_**3**_
**set 3**		
|0〉	**MW**_**2**_	**MW**_**0**_
|−1〉	**MW**_**2**_	**MW**_**0**_
$\sqrt{0.5}(|+1\rangle -|-1\rangle )$	**MW**_**2**_	**MW**_**0**_
$\sqrt{0.5}(|+1\rangle -i|-1\rangle )$	**MW**_**2**_	**MW**_**1**_

NA, not available. A MW_2_ *π* pulse is used to carry out population reversal when eigenvector set 3 is used. Rabi oscillation scheme is then executed by MW channel listed in collum “Rabi”.

### Entropic uncertainty for multiple relations and multiple measurements

Here we summarize the details of several multiple-measurement entropic uncertainty relations being used in the main article. Generally, a multiple-measurement entropic uncertainty relation is of the following form.

2 $$\sum _{m\mathrm{=1}}^{N}H({M}_{m})\ge B({M}_{1},{M}_{2}\,,\mathrm{..}.,\,{M}_{N},\rho ),$$

where {*M*_*m*_} is a set of quantum measurements of cardinality *N* and *B*(⋅) is a non-negative bound which is generally a function of the measurements as well as the density operator *ρ* of the measured system.

For experimental demonstration for entropic uncertainty relations for multiple measurements, we choose to measure three measurement operators in three-dimensional space. Our system, a truly three-level system, of which the quantum states corresponding to *m*_*s*_ = 0, *m*_*s*_ = −1 and *m*_*s*_ = +1 are denoted by |0〉, |−1〉 and |+1〉, respectively. Generally, the entropic uncertainty for the three-measurement case is lower bounded by *B*(*M*_1_, *M*_2_, *M*_3_, *ρ*) which depends on the measurements *M*_1_, *M*_2_ and *M*_3_ and chosen initial states *ρ*. The measurements are chosen with eigenvectors as

3 $$\begin{matrix}{M}_{1} & = & \{|0\rangle ,|-1\rangle ,|+1\rangle \},\\ {M}_{2} & = & \{\sqrt{0.5}(|0\rangle -|+1\rangle ),|-1\rangle ,\sqrt{0.5}(|0\rangle +|+1\rangle )\},\\ {M}_{3} & = & \{\sqrt{a}|0\rangle +\sqrt{b}|-1\rangle ,\sqrt{b}|0\rangle -\sqrt{a}|-1\rangle ,|+1\rangle \},\end{matrix}$$

where *b* = 1 − *a* and *a* ∈ [0, 1] is required. For a detailed comparison, we take three different lower bounds into consideration, which include Rudnicki-Puchala-Zyczkowski (RPZ) direct sum majorization bound^20^, simply constructed bound (SCB) and the recent generalized Maassen-Uffink (MU) bound figured out by Liu, Mu and Fan (LMF)^19^. See Methods for details.

The electron spin of NV center is initialized with a 532 nm laser pulse. Projection measurements with three sets of eigenvectors are used to ensure the initial state of the NV electron spin, then the measurement entropy of each set of eigenvectors is determined. MW pulses of various length, frequencies and phases as shown in Table 2, are employed to carry out the projection. A Rabi oscillation signal is used to read out the result after a projection. The pulse sequence is shown in Fig. 4(d).

**Table 2:** Projection of the Eigenvectors.

Eigenvector	MW channel	MW length
(1 0 0)^1^	**MW**_**0**_	0
(0 1 0)	**MW**_**0**_	*π*
(0 0 1)	**MW**_**2**_	*π*
(0 $\sqrt{0.5}$ $\sqrt{0.5}$)	**MW**_**2**_ **, MW**_**0**_	*π*, 1.5*π*
(0 $\sqrt{0.5}$ $-\sqrt{0.5}$)	**MW**_**2**_ **, MW**_**0**_	*π*, 0.5*π*
($\sqrt{0.1}$ $i\sqrt{0.9}$ 0)	**MW**_**1**_	1.9*π*
($\sqrt{0.9}$ $-i\sqrt{0.1}$ 0)	**MW**_**1**_	0.1*π*
($\sqrt{0.2}$ $i\sqrt{0.8}$ 0)	**MW**_**1**_	1.8*π*
($\sqrt{0.8}$ $-i\sqrt{0.2}$ 0)	**MW**_**1**_	0.2*π*
($\sqrt{0.3}$ $i\sqrt{0.7}$ 0)	**MW**_**1**_	1.7*π*
($\sqrt{0.7}$ $-i\sqrt{0.3}$ 0)	**MW**_**1**_	0.3*π*
($\sqrt{0.4}$ $i\sqrt{0.6}$ 0)	**MW**_**1**_	1.6*π*
($\sqrt{0.6}$ $-i\sqrt{0.4}$ 0)	**MW**_**1**_	0.4*π*
($\sqrt{0.5}$ $i\sqrt{0.5}$ 0)	**MW**_**1**_	1.5*π*
($\sqrt{0.5}$ $-i\sqrt{0.5}$ 0)	**MW**_**1**_	0.5*π*
($\sqrt{0.5}$ 0 $i\sqrt{0.5}$)	**MW**_**2**_	1.5*π*
($\sqrt{0.5}$ 0 $-i\sqrt{0.5}$)	**MW**_**2**_	0.5*π*

^1^The vector (*α*, *β*, *γ*) stands for *α*|0〉 + *β*|−1〉 + *γ*|+1〉. Each projection process is carried out by MW pulses from left to right with MW lengths listed behind.

**Figure 5:**
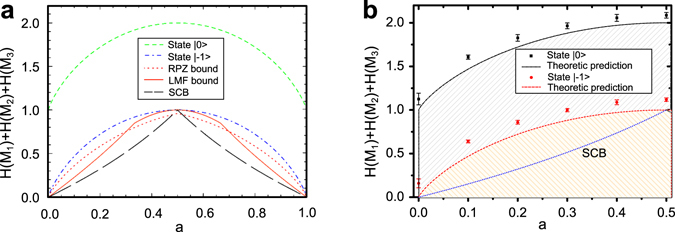
Entropic uncertainty relations for three measurements in the three-dimensional system. (**a**) Comparison between several bounds and entropic uncertainty with respect to *a*, including the maximal SCB (long-dashed black line), RPZ bound (dotted red line) and LMF bound (solid orange line). Dashed green line is for the theoretic result of state |0〉 and dashed-dotted blue line is for that of state |−1〉. (**b**) Comparison between the predicted measurement entropy, experiment results and SCB with respect to parameter *a*. The error bars use the standard error (SE).

Specifically, since entropic uncertainty relations are state dependent, we choose two initial states |0〉 and |−1〉 in our experiment and the theoretic predictions compared with the three kinds of lower bounds are shown in Fig. 5(a). It should be noticed that initial state |−1〉 is proven to have the minimum sum of entropies for the measurements expressed in Eq. (3). The experimental results of the sum of entropies of two initial states with respect to different values of *a* are compared with the theoretic predictions in Fig. 5(b). These results have clearly verified the entropic uncertainty relations predicted by the theory and the lower bounds. The difference between the theoretic prediction and experiment result may be attributed to decoherence of electron spin during the controls and measurements. Since the measured state is initially prepared as a pure state, decoherence will increase the von Neumann entropy of the measured state and enhance the sum of entropic uncertainties. These analyzes can be also manifested by the lower bounds of entropic uncertainty relations discussed in the Methods.

## Discussion

In conclusion, we report the first room-temperature implementation of entropic uncertainty relations for three measurements in a three-dimensional solid-state system: the nitrogen-vacancy center in pure diamond. As summarized in Fig. 5(b), we have experimentally investigated entropic uncertainty relations for multiple measurements with different measured states of spin-1 electron spin of a single NV center and different kinds of three observables. Differing from ordinary used optical systems, our system is a truly three-level system and has overcome the defects of post-selection. The significance of physics for multiple measurements is that the uncertainty principle can be more precisely formulated and demonstrated for a high-dimension quantum state. Differring from the well-studied nonlocality, entanglement or other quantumness of correlations, the uncertainty relations are due to the superposition principle in quantum mechanics. Thus the demanding for physical implementation is that it should be an indivisible quantum system. Our experiment system is naturally three-dimension, and our experimental results confirm the theoretical expectation from the uncertainty relations. Our result may shed new light on the differences between quantum and classical physics in higher-dimension.

## Methods

### State tomography

State tomography is performed by projecting the initial state, denoted by *ρ*, to three sets of eigenvectors indicated in Table 1. Figure 2(b) indicates the pulse sequence of a state tomography measurement. The initial state is prepared by adopting MW_0_ 26 ns and MW_2_ 26 ns to the electron spin of NV center, then Rabi oscillations carried out by various MW channels are used to read out the projection value on each eigenvector (Table 1). Diagonal elements *ρ*_0,0_ = 〈0|*ρ*|0〉, *ρ*_−1,−1_ = 〈−1|*ρ*|−1〉, *ρ*_+1,+1_ = 〈+1|*ρ*|+1〉 are obtained by projection values directly. Non-diagonal elements, for example, *ρ*_−1,0_ = 〈−1|*ρ*|0〉 and *ρ*_0,−1_ = 〈0|*ρ*|−1〉 are solved from a set of equations

4 $${\langle \rho \rangle }_{(|0\rangle -|-1\rangle )}={\rho }_{\mathrm{0,0}}+{\rho }_{-\mathrm{1,}-1}-{\rho }_{\mathrm{0,}-1}-{\rho }_{-\mathrm{1,0}},$$

5 $${\langle \rho \rangle }_{(|0\rangle -i|-1\rangle )}={\rho }_{\mathrm{0,0}}+{\rho }_{-\mathrm{1,}-1}-i{\rho }_{\mathrm{0,}-1}+i{\rho }_{-\mathrm{1,0}}.$$

A *π*-pulse of MW_2_ is used to change the population between *m*_*s*_ = 0 and *m*_*s*_ = +1 in order to get the diagonal and nonagonal elements between *m*_*s*_ = −1 and *m*_*s*_ = +1. The state tomography result of the electron spin superposition state $\frac{1}{\sqrt{3}}(|0\rangle +|-1\rangle +|+1\rangle )$ is

6 $$[\begin{matrix}0.3314 & 0.2977-0.0392i & 0.3200+0.0583i\\ 0.2977+0.0392i & 0.3306 & 0.2460+0.0621i\\ 0.3200-0.0583i & 0.2460-0.0621i & 0.3380\end{matrix}]$$

with which von-Neumann entropy *S*(*ρ*) = −Tr(*ρ*log_2_ *ρ*) can be calculated to be 0.4022 and the fidelity is calculated.

### SCB

From the two-measurement MU inequality as well as the simple relation *H*(*M*_*i*_) ≥ *S*(*ρ*), we can easily obtain a lower bound as

7 $$B=(N-\frac{k}{2})S(\rho )-\frac{1}{2}\,{\rm{l}}{\rm{o}}{\rm{g}}[c({M}_{1},{M}_{2})c({M}_{2},{M}_{3})...\,c({M}_{k},{M}_{1})]$$

where we have 2 ≤ *k* ≤ *N* or *k* = 0 with which we define the second term of the r.h.s. to be zero. We call the maximal value among all bounds deduced in this manner the *simply constructed bound* (SCB), which is explicitly expressed as

8 $${B}_{{\rm{S}}{\rm{C}}{\rm{B}}}=\mathop{max}\limits_{k,\sigma }\{-\frac{1}{2}\,{\rm{l}}{\rm{o}}{\rm{g}}[{C}_{k,\sigma }]+(N-\frac{k}{2})S(\rho )\},$$

where *C*_*k*,*σ*_ := *c*(*M*_*σ*(1)_, *M*_*σ*(2)_)... *c*(*M*_*σ*(*k*)_, *M*_*σ*(1)_). Note that we have considered all possible permutations *σ* among the indices of the measurements.

### LMF’s generalized MU bound

In a recent work^19^ the following lower bound of generalized entropic uncertainty relations for multiple measurements has been proven

9 $${B}_{{\rm{LMF}}}=(N-\mathrm{1)}S(\rho )-\,\mathrm{log}(b),$$

where

10 $$b=\mathop{max}\limits_{{i}_{N}}\{\sum _{{i}_{2}\sim {i}_{N-1}}\mathop{max}\limits_{{i}_{1}}[c({u}_{{i}_{1}}^{1},{u}_{{i}_{2}}^{2})]{{\rm{\Pi }}}_{m=2}^{N-1}c({u}_{{i}_{m}}^{m},{u}_{{i}_{m+1}}^{m+1})\},$$

where $|{u}_{i}^{j}\rangle$ denotes to the *i* th basis vector of the *j* th measurement $c({u}_{m}^{n},{u}_{i}^{j})={|\langle {u}_{m}^{n}|{u}_{i}^{j}\rangle |}^{2}$. We regard this LMF lower bound as a generalization of the MU bound because it explicitly reduces to MU bound if we take *N* = 2. One advantage of this result is that the role of the intrinsic uncertainty of the pre-measurement state has been explicitly demonstrated.

### RPZ direct sum majorization bound

RPZ have introduced an alternative approach to multiple-measurement entropic uncertainty relations^20^. By choosing a certain orthonormal basis in the *d*-dimensional state space, we can rewrite all those basis vectors $\{|{u}_{i}^{j}\rangle \}$ as column vectors in $\mathcal{C}^{d}$. Then define coefficients $\mathcal{S}_{k}$ as follow.

11 $$\mathcal{S}_{k}=\,max\{{\sigma }_{1}^{2}(|{u}_{{i}_{1}}^{{j}_{1}}\rangle,|{u}_{{i}_{2}}^{{j}_{2}}\rangle,\cdots ,|{u}_{{i}_{k+1}}^{{j}_{k+1}}\rangle)\},$$

where ${\sigma }_{1}^{2}(\cdot )$ denotes the square of the largest singular value of a matrix and the maximum ranges over all subsets {(*i*_1_, *j*_1_), (*i*_2_, *j*_2_), …, (*i*_*k*+1_, *j*_*k*+1_)} of cardinality *k* + 1 of the set {1, 2, …, *d*} × {1, 2, …, *N*}. With this definition, a majorization relation as follows can be proven

12 $${\{{p}_{i}^{j}\}}_{i,j\mathrm{=1}}^{d,N}\prec \{\mathcal{S}_{0},\mathcal{S}_{1}-\mathcal{S}_{0},\mathcal{S}_{2}-\mathcal{S}_{1},\cdots \},$$

where ${p}_{i}^{j}$ is the probability of getting the *i* th outcome of the *j* th measurement. This relation leads to the RPZ lower bound

13 $${B}_{{\rm{RPZ}}}=-\sum _{i\mathrm{=1}}^{dN}(\mathcal{S}_{i}-\mathcal{S}_{i-1})\mathrm{log}(\mathcal{S}_{i}-\mathcal{S}_{i-1}).$$
